# Protective Effects of 6-(Methylsulfinyl)hexyl Isothiocyanate on Aβ_1-42_-Induced Cognitive Deficit, Oxidative Stress, Inflammation, and Apoptosis in Mice

**DOI:** 10.3390/ijms19072083

**Published:** 2018-07-18

**Authors:** Fabiana Morroni, Giulia Sita, Agnese Graziosi, Eleonora Turrini, Carmela Fimognari, Andrea Tarozzi, Patrizia Hrelia

**Affiliations:** 1 Department of Pharmacy and Biotechnology, Alma Mater Studiorum—University of Bologna, via Irnerio 48, 40126 Bologna, Italy; giulia.sita2@unibo.it (G.S.); agnese.graziosi2@unibo.it (A.G.); patrizia.hrelia@unibo.it (P.H.); 2 Department for Life Quality Studies, Alma Mater Studiorum—University of Bologna, Corso d’Augusto, 237, 47900 Rimini, Italy; eleonora.turrini@unibo.it (E.T.); carmela.fimognari@unibo.it (C.F.); andrea.tarozzi@unibo.it (A.T.)

**Keywords:** 6-(methylsulfinyl)hexyl isothiocyanate, Aβ oligomers, neuroprotection, oxidative stress, neuroinflammation, Alzheimer’s disease

## Abstract

Alzheimer’s disease (AD) is the most common form of dementia among older people. Although soluble amyloid species are recognized triggers of the disease, no therapeutic approach is able to stop it. 6-(Methylsulfinyl)hexyl isothiocyanate (6-MSITC) is a major bioactive compound in Wasabia japonica, which is a typical Japanese pungent spice. Recently, in vivo and in vitro studies demonstrated that 6-MSITC has several biological properties. The aim of the present study was to investigate the neuroprotective activity of 6-MSITC in a murine AD model, induced by intracerebroventricular injection of β-amyloid oligomers (Aβ_1-42_O). The treatment with 6-MSITC started 1 h after the surgery for the next 10 days. Behavioral analysis showed that 6-MSITC ameliorated Aβ_1-42_O-induced memory impairments. The decrease of glutathione levels and increase of reactive oxygen species in hippocampal tissues following Aβ_1-42_O injection were reduced by 6-MSITC. Moreover, activation of caspases, increase of inflammatory factors, and phosphorylation of ERK and GSK3 were inhibited by 6-MSITC. These results highlighted an interesting neuroprotective activity of 6-MSITC, which was able to restore a physiological oxidative status, interfere positively with Nrf2-pathway, decrease apoptosis and neuroinflammation and contribute to behavioral recovery. Taken together, these findings demonstrated that 6-MSITC could be a promising complement for AD therapy.

## 1. Introduction

Alzheimer’s disease (AD) is a progressive and fatal neurodegenerative disease. It is increasing rapidly in frequency as the population ages and more people enter into the major risk period for this age-related disease. AD is an irreversible disorder that slowly destroys memory and thinking skills and, eventually, the ability to carry out the simplest tasks. The success rate of drug development for AD has been poor and there is an urgent need to develop new treatments for the disease [1]. Attempts to find cures for AD have failed so far, in spite of intellectual and financial efforts. The failure of the therapeutic strategies to impact the natural course of the disease may reflect the complexity of AD and the lack of understanding of its exact etiopathogenesis. 

Amyloid β-peptide (Aβ) is crucially involved in AD as the main component of the amyloid (senile) plaques found in the brains of Alzheimer’s patients. The amyloid cascade hypothesis, which posits that the deposition of the Aβ peptide in the brain is a crucial event in AD pathology, was originally proposed in 1992. According to it, the cause of the disease is Aβ in the form of large aggregates (plaques) [2] or, more recently, in the form of soluble oligomeric species [3]. The amyloid cascade hypothesis has since its formulation been subject to some criticism, but has ever since remained the predominant hypothesis for the identification of potential therapeutic strategies. Several studies have suggested that Aβ can diffuse readily through the brain parenchyma and activate a cascade of pathogenic events, such as the culmination of neuronal apoptosis/necrosis, and induction of oxidative stress and neuroinflammation in the cortex and hippocampus, which are two of the affected brain regions in AD [4,5,6].

**Figure 1:**
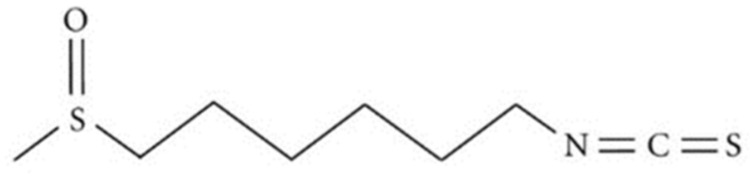
Chemical structure of 6-(methylsulfinyl)hexyl isothiocyanate (6-MSITC).

Given the many plausible limitations of targeted Aβ-based therapies, it is likely that effective AD therapeutics will include parallel strategies that confer neuroprotection against Aβ and other agents and processes (i.e., oxidative stress and inflammation) causing neuronal dysfunction and degeneration. In addition to strategies designed to decrease Aβ levels, it is possible that successful therapeutic strategies will require the concomitant application of neuroprotective agents [7]. In the context of neuroprotection, isothiocyanates (ITCs) have already shown interesting effects at the Central Nervous System (CNS) level [8,9,10]. They consist of compounds resulting from the reaction between glucosinolates and the endogenous enzyme myrosinase in cruciferous vegetables that occur when tissues are damaged [11]. Accumulating evidence suggests that ITCs exert their effects through a variety of signaling pathways involved in detoxification, inflammation, and apoptosis, among others [12,13,14]. In this study, we have focused on an aromatic component of the Japanese spice, wasabi. Wasabi (Wasabia japonica) belongs to the Brassicaceae family and it is characterized by a high concentration of ITCs, predominantly long-chain ITCs [15]. Among the bioactive components of wasabi, several ITC analogues have been identified, and 6-(methylsulfinyl)hexyl isothiocyanate (6-MSITC) (Figure 1) represents the major active compound. 

Several studies demonstrated the pharmacological potencies of 6-MSITC, such as antiplatelet [16], antimicrobial [17], anti-inflammatory [18,19], and anticancer [20,21] effects. It is known that ITCs are primarily metabolized through the mercapturic acid pathway in vivo [22]. ITCs appear to penetrate the cellular membrane by diffusion and rapidly conjugate with intracellular reduced glutathione (GSH) via their ITC group (–N=C=S). The methylsulfinyl group (CH_3_–S(=O)–) and the length of alkyl chain of 6-MSITC might contribute to cell membrane permeability [23]. We previously reported the neuroprotective effects of 6-MSITC on behavioral and biochemical alterations in mice caused by striatal 6-hydroxydopamine injection by regulating the GSH-dependent antioxidant systems [24]. In this paper, we describe the potential neuroprotective activity of 6-MSITC in a murine AD model obtained by intracerebroventricular (i.c.v.) injection of Aβ_1-42_ oligomers (Aβ_1-42_O) and discuss the molecular mechanisms with special attention to its antioxidant and anti-inflammatory properties. To the best of our knowledge, this is the first study of the effect of 6-MSITC on Aβ-induced toxicity.

## 2. Results

### 2.1. Effects of 6-MSITC on the Cognitive Functions After Aβ_1-42_O Injection

**Figure 2:**
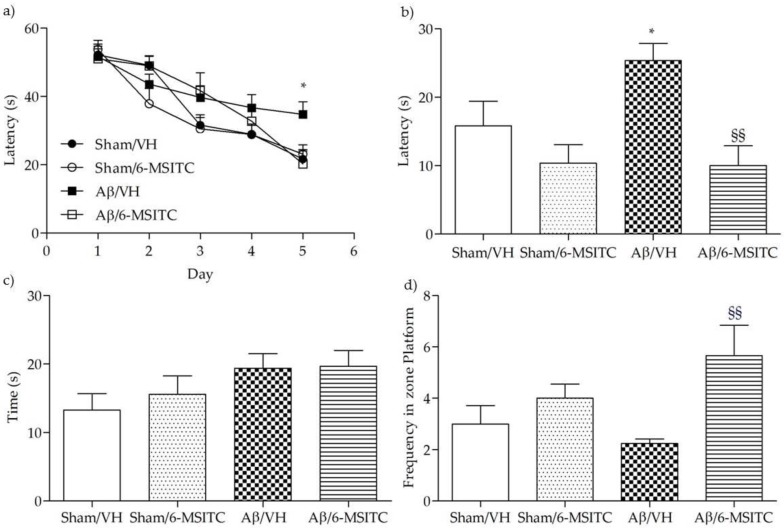
Effects of 6-MSITC (5 mg/kg) on the performance in the training (**a**) and probe trials (**b**–**d**) of the Morris Water Maze (MWM) test in Aβ_1-42_O-injected mice. The training trials were carried out for 5 days (four per day), the probe trial was performed on day 6. Escape latency (**b**), time spent in the opposite quadrant to the platform zone (**c**), and frequency in the platform zone (**d**) were recorded in the probe test. Values are expressed as mean ± SEM (*n* = 10) (**a**: * *p* < 0.05 vs. Sham/VH group; **b**: * *p* < 0.05 vs. Sham/VH group, ^§§^ *p* < 0.01 vs. Aβ/VH; **d**: ^§§^ *p* < 0.01 vs. Aβ/VH; ANOVA, post hoc test Bonferroni).

To assess the protective effect of 6-MSITC against Aβ_1-42_O-induced spatial memory deficits in mice, we evaluated the behavioral performance of the mice using the Morris Water Maze (MWM) test. At the last day of training, the escape latency was significantly different among various groups. The mice that received intraperitoneal (i.p.) injection of vehicle after the lesion induced by i.c.v. injection of Aβ_1−42_O (Aβ/VH group) took significantly more time to find the hidden platform as compared to the sham group, confirming that Aβ_1−42_O could induce impairments of spatial learning in mice (*p* < 0.05, Figure 2a). Moreover, the mice treated with 6-MSITC (Aβ/6-MSITC group) spent a significantly shorter time finding the hidden platform as compared to the Aβ/VH group, indicating that 6-MSITC could attenuate Aβ_1−42_O-induced impairments of spatial learning. In terms of swimming speed, there was no significant difference among different groups throughout the five training days. In the probe trial, the platform was removed, and mice were allowed to swim freely. The mice in the Aβ/VH group took more time to reach the platform location as compared to the sham group, suggesting that Aβ_1−42_O also caused impairments of spatial memory (*p* < 0.05, Figure 2b). Interestingly, 6-MSITC significantly reversed Aβ_1−42_O-induced impairments of spatial memory in mice, as demonstrated by the decrease of the escape latency and by the increase of the frequency in the platform zone as compared to the Aβ/VH group (*p* < 0.01, Figure 2b,d).

**Figure 3:**
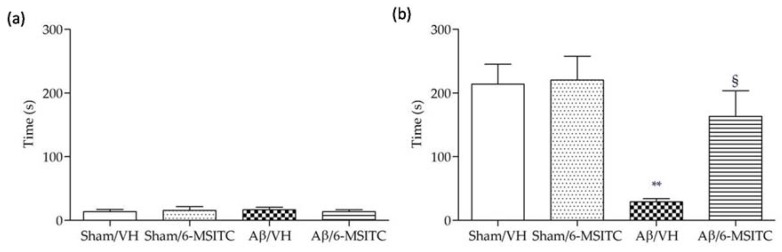
Effects of 6-MSITC (5 mg/kg) on the performance in the training (**a**) and passive avoidance test (**b**) in Aβ_1-42_O-injected mice. The latency time for entering in the compartment of preference was recorded. Values are expressed as mean ± SEM (*n* = 10) (** *p* < 0.01 vs. Sham/VH group, ^§^ *p* < 0.01 vs. Aβ/VH; ANOVA, post hoc test Bonferroni).

Following the completion of the MWM test, we performed the passive avoidance test to evaluate fear-motivated hippocampal memory. As we know, mice instinctively prefer the dark compartment. If an electric shock is given, a fear memory will be subsequently formed to delay the mice to reenter the dark compartment. Greater response latency in the retention phase indicates better hippocampal memory. As shown in Figure 3a, latencies of mice from different groups did not show significant difference on the training day, underlying there were no distinct preferences to escape into the dark compartment among mice with different treatments. However, after electric shock was delivered on the probe day 24 h later, Aβ/VH mice showed significantly decreased latency time compared with sham groups (*p* < 0.01, Figure 3b), suggesting an apparent memory deficit was caused after Aβ insult. By contrast, mice treated with 6-MSITC displayed a robust latency elevation, which confirmed 6-MSITC treatment can remarkably improve Aβ_1-42_O-induced memory deficit (*p* < 0.05, Figure 3b).

### 2.2. Effects of 6-MSITC on Hippocampal Cell Death After Aβ_1-42_O Injection

**Figure 4:**
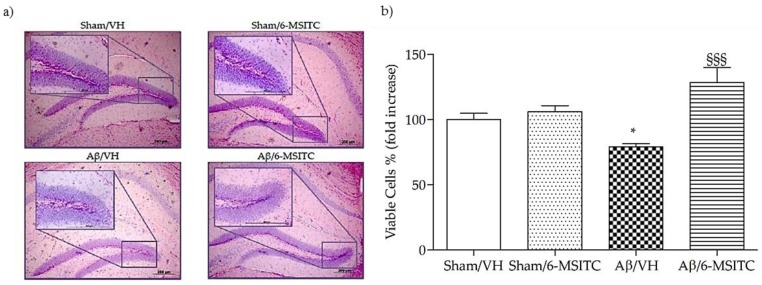
Effects of 6-MSITC (5 mg/kg) on neuronal cell death 20 days after Aβ_1-42_O injection. Representative H&E staining of coronal sections containing the hippocampus. Magnification 10× and 40×, scale bar 200 μm (**a**). Quantitative analysis of H&E staining (**b**). Values are expressed as mean of fold increase ± SEM (*n* = 10) of the density of each experimental group compared to Sham/VH group (**b**: * *p* < 0.05 vs. Sham/VH, ^§§§^ *p* < 0.001 vs. Aβ/VH; ANOVA, post hoc test Bonferroni).

We investigated the neuroprotective effect of 6-MSITC on Aβ_1−42_O-induced hippocampal cell death using Hematoxylin-Eosin (H&E) staining. After behavioral analysis, mice were sacrificed and a significant decrease in the density of healthy neurons in CA1 region of hippocampus was observed in the Aβ/VH group compared with the sham mice (*p* < 0.05, Figure 4b). As expected, typical neuropathological changes, including neuron loss and nucleus shrinkage or disappearance, were found in the CA1 of hippocampus in Aβ_1−42_O-injected mice. Neuronal injuries were markedly less severe after treatment with 6-MSITC (Figure 4a), and the number of injured neurons in the Aβ/6-MSITC group was significantly lower than in the Aβ/VH group (*p* < 0.001, Figure 4b).

**Figure 5:**
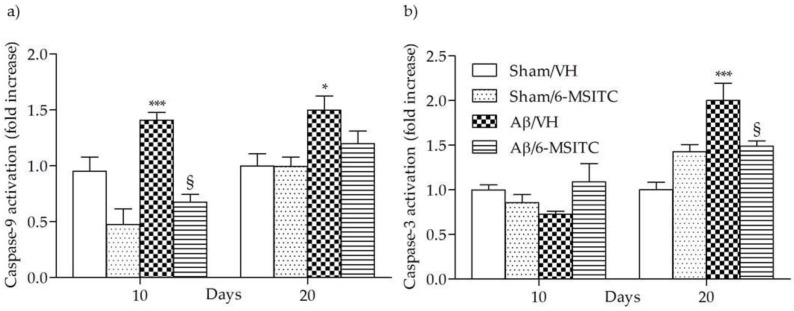
Effects of 6-MSITC (5 mg/kg) on caspase-9 (**a**) and -3 (**b**) activations 10 and 20 days after Aβ_1-42_O injection. Caspase-9 and -3 activations were determined using a specific chromogenic substrate in hippocampal samples. Values are expressed as mean of fold increase ± SEM (*n* = 10) of optical density (OD) of each experimental group compared to the Sham/VH group (**a**: * *p* < 0.05 and *** *p* < 0.001 vs. sham groups, ^§^ *p* < 0.05 vs. Aβ/VH; **b**: *** *p* < 0.001 vs. Sham/VH, ^§^ *p* < 0.05 vs. Aβ/VH; ANOVA, post hoc test Bonferroni).

Caspase activation in the brain has been reported to be part of AD neuropathogenesis and to contribute to cognitive dysfunction [25]. Caspase-9 and -3 are known as biomarkers of oxidative stress-induced cell death, which is mediated by the mitochondria-dependent apoptotic pathway [26]. The activated caspase-9 subsequently cleaves and activates the downstream effector procaspase-3, contributing to caspase-dependent apoptosis. As shown in Figure 5, the activation of caspase-9 and -3 were increased significantly in the hippocampal samples of Aβ_1-42_O-treated group, when compared to the sham group, in particular caspase-9 activity reached a maximum at 10 days post-Aβ_1-42_O-injection and caspase-3 at 20 days (*p* < 0.001). However, 6-MSITC treatment was shown to be effective at inhibiting activation of both caspases induced by Aβ_1-42_O (*p* < 0.05).

### 2.3. Effects of 6-MSITC on Oxidative Stress After Aβ_1-42_O Injection

**Figure 6:**
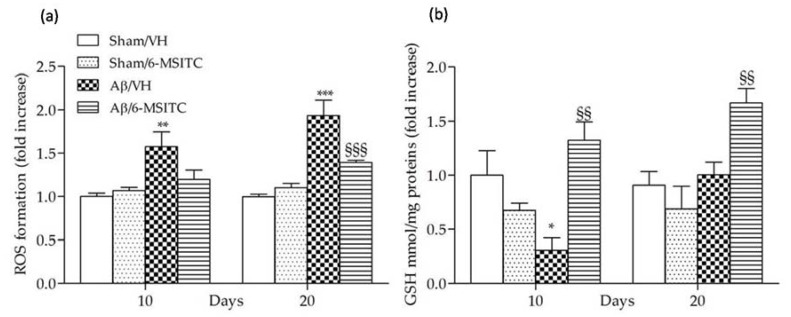
Effects of 6-MSITC (5 mg/kg) on cellular redox status after Aβ_1-42_O injection. Redox status was determined in hippocampal samples 10 and 20 days after Aβ_1-42_O injection (**a**) based on fluorescence emission of 2′,7′-dichlorofluorescein (DCF) at 535 nm after excitation at 485 nm. Values are expressed as mean of fold increase ± SEM (*n* = 10) of fluorescence intensity arbitrary units (UF) of each experimental group compared to the Sham/VH group. GSH content was measured using a colorimetric assay in hippocampal samples 10 and 20 days after Aβ_1-42_O injection (**b**). Values are calculated using a standard calibration curve and expressed as mean of fold increase ± SEM (*n* = 10) of mmol GSH/mg proteins compared to the Sham/VH group. (**a**: ** *p* < 0.01 and *** *p* < 0.001 vs. sham groups, ^§§§^ *p* < 0.001 vs. Aβ/VH group; **b**: * *p* < 0.05 vs. Sham/VH, ^§§^ *p* < 0.01 vs. Aβ/VH groups; ANOVA, post hoc test Bonferroni).

Evidence indicates that Aβ enters the mitochondria and induces reactive oxygen species (ROS) formation and oxidative stress in the early stages of AD pathogenesis, and ROS generation may be involved in senile plaque accumulation in AD brains [27,28]. As previously reported, Aβ_1−42_O-injection causes oxidant stress to the mice brain [29], indicated by significant increased ROS formation in the hippocampal samples. We found that 6-MSITC treatment resulted in a drastic reduction of ROS compared with Aβ/VH group (*p* < 0.001, 20 days after Aβ_1-42_O-injection, Figure 6a), demonstrating that the protective effect of 6-MSITC against Aβ_1-42_O-induced oxidative stress was because of its inhibition of excessive ROS generation by the Aβ insult. Cellular antioxidants such as GSH detoxify oxidative stress-induced damage. GSH, the most abundant endogenous antioxidant, plays a crucial role in detoxification of ROS and regulation of intracellular redox environment [30]. Brain cells are vulnerable to oxidative stress; therefore, GSH level regulation may contribute to new treatment strategy development. We here showed that GSH levels were decreased in the hippocampus of Aβ-treated mice (*p* < 0.05, 10 days after Aβ_1-42_O-injection, Figure 6b), supporting previous reports that intrahippocampal Aβ injection could lead to oxidative stress and antioxidant defense reduction [31]. However, 6-MSITC treatment induced a complete restoration of the GSH levels depleted by Aβ_1-42_O treatment (*p* < 0.01). 

**Figure 7:**
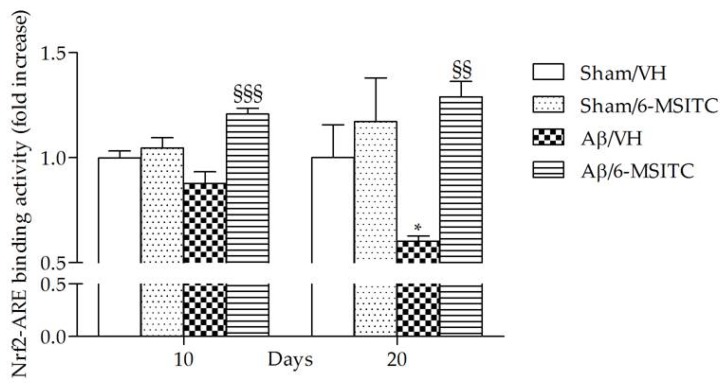
Effects of 6-MSITC (5 mg/kg) on Nrf2 activation after Aβ_1-42_O injection. Nrf2 activation was detected 10 and 20 days after Aβ_1-42_O injection using an Nrf2-based ELISA kit on nuclear extract of hippocampal samples. Values are expressed as mean of fold increase ± SEM (*n* = 10) of the optical density (OD) of each group compared to the Sham/VH group. (* *p* < 0.05 vs. sham groups, ^§§^ *p* < 0.01 and ^§§§^ *p* < 0.001 vs. Aβ/VH group; ANOVA, post hoc test Bonferroni).

ITCs are unique indirect antioxidants that enhance the antioxidant barrier of the organism, and activate the nuclear factor E2-related factor 2 (Nrf2)-antioxidant responsive element (ARE) pathway [9]. The Nrf2-ARE pathway involves several antioxidative and anti-inflammatory genes that remove oxidized proteins and prevent/remove Aβ protein depositions [32]. We then examined whether 6-MSITC treatment could alter the Nrf2 activation in the hippocampus of mice. ELISA analysis indicated that Nrf2 DNA-binding activities in nuclear fractions from the Aβ/6-MSITC mice were significantly increased compared with the Aβ/VH group (*p* < 0.001 and *p* < 0.01, 10 and 20 days post-injection, respectively; Figure 7). Thus, 6-MSITC was able to restore the Nrf2 binding activity inhibited by Aβ_1-42_O treatment.

### 2.4. Effects of 6-MSITC on ERK1/2 and GSK3β Activities After Aβ_1-42_O Injection

**Figure 8:**
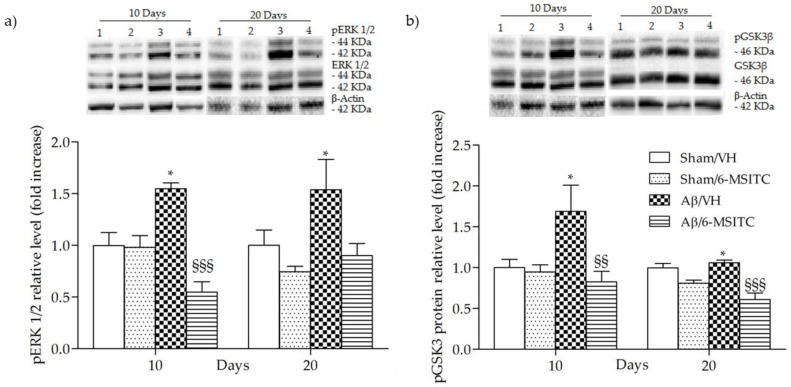
Effects of 6-MSITC (5 mg/kg) on ERK (pERK) and GSK3β phosphorylations (pGSK3β) after Aβ_1-42_O injection. pERK, pGSK3β and β-actin were determined 10 and 20 days after Aβ_1-42_O injection by Western Blotting at 44–42 kDa (**a**) and at 46 kDa (**b**) using respectively total ERK, total GSK3β and β-actin as loading control. Top: representative images of the protein expression in the hippocampus. Numbers indicate animal groups as follows: 1 Sham/VH, 2 Sham/6-MSITC, 3 Aβ/VH, 4 Aβ/6-MSITC. Bottom: quantitative analysis of the Western Blotting results for the pERK and pGSK3β levels. The graphs show densitometry analysis of the bands appertaining to the protein of interest. Values are expressed as mean of fold increase ± SEM (*n* = 10) of each group compared to the Sham/VH group (**a**: * *p* < 0.05 vs. sham groups, ^§§§^ *p* < 0.001 vs. Aβ/VH group; **b**: * *p* < 0.05 vs. sham groups, ^§§^ *p* < 0.01 and ^§§§^ *p* < 0.001 vs. Aβ/VH group; ANOVA, post hoc test Bonferroni).

The mitogen-activated protein kinase (MAPK) signaling pathway is engaged in the transcriptional regulation of neuronal apoptosis and has been shown to play a key role in AD [33], therefore the phosphorylation of extracellular signal-regulated protein kinases 1 and 2 (ERK1/2), a member of MAPK family, was also detected in the present study. Data showed that Aβ_1-42_O-injection aroused the phosphorylation of ERK1/2 compared with the sham group (*p* < 0.05, Figure 8a). However, treatment with 6-MSITC remarkably repressed the phosphorylation of ERK1/2 induced by Aβ_1-42_O (*p* < 0.001, 10 days post-injection, Figure 8a).

Increasing evidence suggests that the glycogen synthase kinase 3 (GSK3) activity is directly impacted by Aβ_1−42_O exposure and it is altered in AD brains [34]. To explore the effects of 6-MSITC treatment on GSK3 activity, the amount of its phosphorylation was assessed by western blot studies. In agreement with the data from our previous studies [6,29], Aβ_1-42_O induced a significant increase (*p* < 0.05) of GSK3 phosphorylation on Ser9, which corresponds to its inactivity, as compared to sham mice (Figure 8b). 6-MSITC treatment reversed the effects of Aβ_1-42_O and significantly decreased (*p* < 0.01 and *p* < 0.001, 10 and 20 days post-injection) GSK3β inhibitory phosphorylation.

### 2.5. Effects of 6-MSITC on Neuroinflammation After Aβ_1-42_O Injection

**Figure 9:**
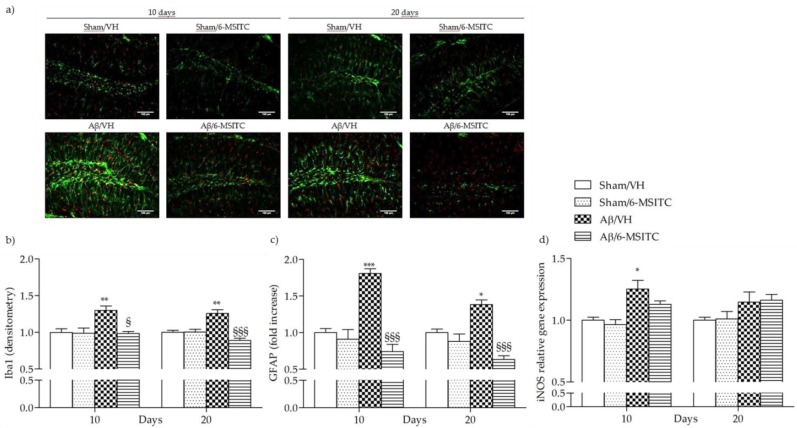
Effects of 6-MSITC (5 mg/kg) on inflammatory response after Aβ_1-42_O injection. Representative photomicrographs (**a**) of immunostaining for GFAP (green) and Iba1 (red) at 10 and 20 days post Aβ_1-42_O injection in brain coronal sections containing hippocampal structure. Magnification 20×, scale bar 100 μm. Quantitative analysis of GFAP (**b**) and Iba1 (**c**) immunostaining. Values are expressed as mean of fold increase ± SEM (*n* = 10) of the fluorescent intensity of each experimental group compared to the Sham/VH group. iNOS mRNA relative expression (**d**) was determined in hippocampal samples 10 and 20 days after Aβ_1-42_O injection through the 2^−ΔΔCt^ method (**b**: ** *p* < 0.01 vs. sham groups, ^§^ *p* < 0.05 and ^§§§^ *p* < 0.001 vs. Aβ/VH group; **c**: * *p* < 0.05 and *** *p* < 0.001 vs. sham groups, ^§§§^ *p* < 0.001 vs. Aβ/VH group; **d**: * *p* < 0.05 vs. sham group; ANOVA, post hoc test Bonferroni).

The early stages of AD are characterized by neuroinflammatory reactions mediated by microglia and astrocytes activation that play a key role in the pathology evolution. Several studies have demonstrated that neurons could amplify local inflammatory reactions by producing mediators and act as an important contributor in the pathogenesis of AD [35]. Activated astrocytes and microglia facilitate Aβ clearance, but also mediate inflammation via production of proinflammatory cytokines and immunostimulatory molecules [36]. Glial fibrillary acidic protein (GFAP) and ionized calcium-binding adaptor molecule 1 (Iba-1) are specific markers for activated astrocytes and microglia, respectively. Immunohistochemical staining for the microgliosis (Iba1) and astrogliosis (GFAP) in hippocampal regions revealed a significant increase in the number of Iba-1 (*p* < 0.01) and GFAP (*p* < 0.001 at 10 days and *p* < 0.05 at 20 days) reactive cells in the Aβ/VH group compared to sham mice. On the other hand, 6-MSITC treatment significantly decreased the number of reactive Iba-1 (*p* < 0.001) and GFAP (*p* < 0.05 at 10 days and *p* < 0.001 at 20 days) cells in the hippocampus of Aβ_1-42_O treated mice (Figure 9a–c), which returned to levels similar to those in the sham group. Activated myeloid cells promote release of nitric oxide (NO) through the activation of inducible NO synthase, iNOS, a hallmark of the classically activated pro*-*inflammatory phenotype [37]. As shown in the Figure 9d, Aβ_1-42_O significantly increased the transcription of the pro-inflammatory factor gene iNOS at 10 days post-injection as compared with the sham group. Treatment with 6-MSITC showed a sizable reduction in iNOS expression, but it was not significant.

## 3. Discussion

The present study is the first to provide evidence that 6-MSITC attenuates Aβ_1-42_O-induced memory impairment, oxidative stress, neuroinflammation, and hippocampal neuronal degeneration in a mouse Aβ_1-42_ model. The i.c.v. Aβ-injection model is a useful complement to transgenic mouse models [38] for the development and evaluation of therapeutic approaches to AD pathology because the mechanisms underlying many characteristics of AD, neuroinflammation, synaptotoxicity, apoptosis and neurodegeneration, remain elusive. However, it is necessary to consider that this acute model unfortunately does not reproduce the gradual rise in Aβ occurring over many years in humans and it is unlikely that it replicates the chronic AD phenotype. However, the use of acute injections may help to clarify how Aβ impairs specific signaling pathways leading to synaptic and memory dysfunctions, and this is crucial when designing new therapeutic strategies.

Moreover, the i.c.v. Aβ-injection model facilitates behavioral studies in a relatively short timeframe. 

Since AD slowly destroys memory and learning skills, we investigated behavioral changes in Aβ_1-42_O-treated mice using the MWM and the passive avoidance tests. As previously reported [6,29], i.c.v. injection of Aβ_1-42_O caused impairments in learning and memory performance of the mice. Interestingly, in the current study, i.p. administration of 6-MSITC significantly attenuated Aβ_1-42_O-induced learning and memory impairments, increasing avoidance retention time in the passive avoidance test and decreasing escape latency and retention of spatial memory in the MWM test. General motor functions were not affected. By histological examination, 6-MSITC administration prevented Aβ_1-42_O-induced neuronal death in the hippocampus. The apoptotic markers found in the postmortem AD brain, such as increased caspase activities, bcl-2 family protein expression, and DNA fragmentations [39] corroborate the hypothesis that Aβ_1-42_O induces neuronal cell death through the apoptotic pathways. Aβ_1-42_O has been shown to activate caspases accompanied by p53 activation. Caspase activation in response to Aβ injection has been implicated in the biochemical cascade during the final stage of apoptosis [40]. Our experimental paradigm also revealed that Aβ_1-42_O activated caspases; as expected caspase-9 activation occurred earlier than caspase-3. Indeed, caspase-9 can activate caspase-3, which can specifically cleave DNA, and inactivate the poly ADP ribose polymerase (PARP) and DNA-dependent protein kinase (DNA-PK) involved in the course of DNA repair after damage. Then, the chromatin is condensed and the ribozyme is activated, and cell apoptosis can occur. And in turn, caspase-3 can activate caspase-9, and they form a positive feedback [41]. Intriguingly, caspase-9 and -3 activations were all attenuated by 6-MSITC treatment. These results were consistent with the anti-apoptotic effects of 6-MSITC shown in our previous in vivo study [24], suggesting that 6-MSITC is able to inhibit the Aβ-induced activation of apoptotic pathway.

All multifactorial pathological changes caused by Aβ oligomers are evidenced to link with oxidative stress and inflammation, and both can lead to neuronal injury [42,43]. Therefore, we hypothesized that the alleviation of oxidative stress and inflammation may attenuate the toxicity of Aβ_1-42_O. GSH is a key intracellular antioxidant that protects against endogenous oxygen radicals. GSH scavenges ROS by directly reacting with them and prevents hydrogen peroxide-induced hydroxyl radical formation. Therefore, GSH level parallels the antioxidant defense capacity and it is a first indicator for oxidative stress in the brain [44]. In this study, the significantly higher ROS levels and the GSH marked lower in the Aβ/VH group further demonstrated that Aβ_1-42_O induced oxidative stress and the antioxidant defenses were not able to offset the oxidant. On the other hand, 6-MSITC decreased ROS levels as well as restored GSH content in Aβ_1–42_O -treated mice, which was probably due to its capacity to modulate the Nrf2 signaling system. The main endogenous defense against oxidative stress involves the Nrf2-dependent antioxidant and anti-inflammatory responses, which involve the activation of Nrf2 and the subsequent increased expression of the downstream cytoprotective proteins [45]. Branca et al. [46] used a genetic approach to remove the Nrf2 gene from APP/PS1 mice, and found that the lack of Nrf2 significantly exacerbates cognitive deficits in APP/PS1, suggesting a clear link between Nrf2 and AD-mediated cognitive decline and further strengthens the connection between Nrf2 and AD. Moreover, in vitro and in vivo studies demonstrated that Nrf2 pathway activation by pharmacological activators plays a protective role against Aβ-induced toxicity [47,48,49]. Consistently, here, 6-MSITC-treated mice showed an abundant accumulation of nuclear Nrf2, along with a reversal of the increased ROS levels, indicating that 6-MSITC activated a potent Nrf2-mediated adaptive response, which is impaired in Aβ-lesioned mice. In accordance with our data, Trio et al. designed a genome-wide DNA microarray analysis of Wasabi-derived ITCs in a neuronal cell model. Among the three ITCs analyzed, 6-MSITC had the strongest regulation on gene expression and most of the genes targeted belonged to oxidative stress response cluster. Specifically, 6-MSITC could stimulate Nrf2-mediated gene expressions through the stabilization of the Nrf2 protein at the post-transcriptional level [50]. Moreover, Hou et al. demonstrated that 6-MSITC enhanced Nrf2/ARE-driven NAD(P)H:quinone oxidoreductase 1 expression by stabilizing Nrf2, which was accomplished by modifying the Kelch-like ECH-associated protein 1 (Keap1) with consequent inhibition of the ubiquitination and proteasomal turnover of Nrf2 [51].

Studies on postmortem human brains indicated the Ras-MAPK pathway as an early driver of AD pathology development [52,53]. It has been proved that the phosphorylation of ERK1/2 contributes to the apoptosis response in neurons [54,55]. Thus, blocking its phosphorylation generates the subsequent inhibition of apoptosis through the regulation of caspase-3 activity [56,57]. On the other hand, overwhelming data shows that the activation of the ERK pathway could revert learning and memory impairments in AD due to the engagement of survival mechanisms [58,59]. According to the contradictory reports on how the ERK signaling pathway may be modulated in AD, for instance in the reversal of cognitive impairment, we think that the activation of ERK needs an appropriate time window to achieve a given benefit. Our results have revealed that enhanced hippocampal caspase activation is associated with phosphorylation of ERK1/2, reinforcing the correlation of the MAPK pathway and neuronal apoptosis and its importance in the pathogenesis of AD. Even more interesting, 6-MSITC treatment is able to reverse the phosphorylation of ERK1/2, by which the activation of apoptotic responses is repressed, supporting the potential neuroprotective activity of 6-MSITC.

Recently, there has been great interest in GSK3 as a potential target for the treatment of AD [34,60]. GSK3β has been implicated in tau hyperphosphorylation, subsequent neurodegeneration [61], and silencing GSK3β leads to reduced plaque and tangle formation in transgenic mouse models of AD [62]. The mechanism by which GSK3β regulates neurodegeneration in AD is only partly understood and direct evidence for this is still limited at present, indeed some studies found no change in GSK3 activity [63] or reduced GSK3 activity [64] in AD. Paradoxical findings can be attributed to an incomplete understanding of a system rather than being manifestations of incongruent properties. Beurel and Jope accurately reviewed that GSK3 controversial effects are most likely the result of the opposite effects on the two major apoptotic signaling pathways and the activity of GSK3 finely contributes to shifts in the balance between survival and apoptosis [65]. Interestingly, GSK3β phosphorylation (Ser9) was associated with up-regulation of antioxidant enzymes, before occurrence of irreversible damage and death [66]. Our results showed that 6-MSITC treatment reversed the effects of Aβ_1-42_O and significantly decreased GSK3β inhibitory phosphorylation. We hypothesize that one possible function of this GSK3β activation induced by 6-MSITC is to alert defenses against redox instability and coordinated redox changes.

Several experiments stated that massive astrocytic activation is involved in the AD pathogenesis leading to the release of various neurotoxic agents with increased GFAP expression, a marker for astrogliosis [67]. Microglia respond quickly to any injury or insult than astrocytes [68] and Iba-1 synthesis occurs only in microglial cells. The elevated expression of Iba-1 and GFAP, observed in our study, clearly demonstrated the activation of macro- and microglial cells after Aβ_1-42_O treatment, but 6-MSITC administration nullifies the active gliosis by diminishing the Iba-1 and GFAP expressions. The synthesis and release of NO via iNOS from astrocytes and microglia is another way in which neuroinflammation can directly influence neuronal apoptosis [69]. Our data confirmed the influence of NO pathway in Aβ toxicity, as demonstrated by the significant increase of iNOS expression in the hippocampus, and 6-MSITC attenuated this effect. Interestingly, Uto et al. showed that 6-MSITC inhibited several inflammatory factors, such as cyclooxygenase-2 (COX-2), iNOS, and inflammatory cytokines at the transcription factor/promoter levels. According to our findings, they demonstrated that 6-MSITC attenuates iNOS expression mainly by blocking c-Jun N-terminal kinases (JNK)-mediated the activator protein-1 (AP-1) activation in lipopolysaccharide-activated murine macrophages [70]. 

In summary, we report here for the first time that 6-MSITC counteracted Aβ_1-42_O neurotoxicity in mice. These results underlined an interesting neuroprotective activity of 6-MSITC, which decreased apoptosis and neuroinflammation, restored physiological oxidative status and interfered positively with the Nrf2-pathway, that led to a significant behavioral recovery in our AD model. These data are promising, however further experimental studies are needed to confirm the 6-MSITC mechanism of action, evaluate its short- and long-term effects, and assess its combination with other therapeutics.

## 4. Materials and Methods

### 4.1. Animals

Nine weeks old male C57Bl/6 mice (25–30 g body weight at the beginning of the experiment; Harlan, Milan, Italy) were housed under 12h light/12h dark cycle (lights on from 7:00 a.m. to 7:00 p.m.) with free access to food and water in a temperature- and humidity-controlled room. All experiments were conducted in accordance with the guidelines set by the European Community Council Directives 86/609/EEC and 2010/63/EU, and the protocols were approved by the corresponding committee at the University of Bologna (PROT. n. IX/77 2013, 26 November 2013). Care was taken to limit their suffering and minimize the number of experimental animals. Mice were allowed to acclimatize for at least one week before the experiments.

### 4.2. Experimental Design

**Figure 10:**
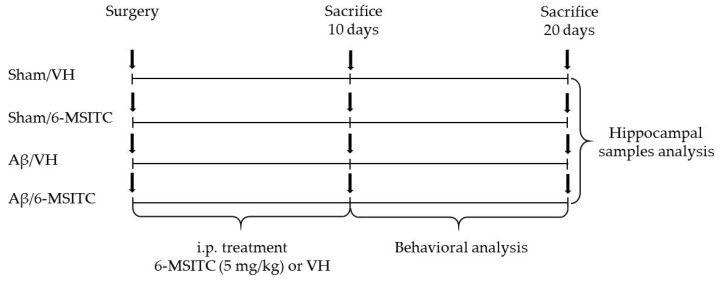
Experimental protocol and 6-MSITC treatment schedule. Mice received i.p. injection of 6-MSITC (5 mg/kg) for 10 days. Animals were sacrificed 10 or 20 days after Aβ_1–42_O injection.

The experimental protocol was based on the stereotaxic i.c.v. injection of Aβ_1-42_O. Animals were randomly divided into four major groups (*n* = 20/group) as follows: Aβ/VH; Aβ/6-MSITC; Sham/VH; Sham/6-MSITC. Two groups received an i.c.v. injection of Aβ_1-42_O, while the other two received the same amount of saline solution (sham groups). One hour after brain lesion, we started i.p. administration of 5 mg/kg of 6-MSITC (Lkt Laboratories, St. Paul, MN, USA) or vehicle (VH, saline) in both lesioned and sham mice. The dose injected was selected on the basis of previous studies [24]. We injected mice everyday once a day for 10 days. At the end of the treatment half the group was sacrificed to proceed with biomolecular analysis while the other animals underwent behavioral assessment before the sacrifice (20 days post-injection). Animals were deeply anesthetized and sacrificed by cervical dislocation to perform immunohistochemistry, neurochemical, and molecular analysis (for the experimental design see Figure 10).

### 4.3. Aβ_1-42_O Preparation and Injection

Aβ_1–42_ peptides (AnaSpec, Fremont, CA, USA) were first dissolved in hexafluoroisopropanol to 1 mg/mL, sonicated, incubated at room temperature for 24 h and lyophilized. The resulting unaggregated Aβ_1–42_ film was dissolved with sterile dimethylsulfoxide to a final concentration of 1 mM and stored at −20 °C until use. The Aβ_1–42_ oligomers were prepared as already described by Tarozzi et al. [71]. Briefly, Aβ_1-42_ stock was diluted into PBS at 40 μM and incubated at 4 °C for 48 h to enhance oligomer formation [72,73]. The day of the surgery, 6 µL of Aβ_1-42_O (40 μM) were injected i.c.v., using a stereotaxic mouse frame (myNeuroLab, Leica-Microsystems Co, St. Louis, MO, USA). Sham mice received the corresponding volume of vehicle into the ventricle. The injection was performed at the following co-ordinates: AP: +0.22, ML: +1.0, DV: −2.5, with a flat skull position. 

### 4.4. Behavioral Analysis

All tests were carried out between 9 a.m. and 4 p.m. One hour before the test, animals were transferred to the experimental room in order to let them acclimatize to the environment. All scores were assigned by the same operator, who was unaware of the animal treatment.

### 4.5. MWM Test

Spatial learning-memory ability was assessed by the MWM test, performed as previously described [6]. The water maze equipment consisted of a circular tank (1.0 m diameter, 50 cm height) filled with water and milk at 22 °C, a hidden platform, and a recording system. The pool was spatially divided into four imaginary quadrants by a computerized tracking/image analyzer system (EthoVision, Noldus, The Netherlands). A circular transparent escape platform was positioned 1.5 cm below the opaque water surface in the middle of the target quadrant. Mice were given orientation navigation tests for five consecutive days. Before the measurement, mice were trained once to find the platform. For each training trial, the mice were randomly dropped into the pool at one of the four positions. The escape latency and the swim path tracking until the mice landed on the platform were recorded on video tape. If the mouse failed to locate the platform within 60 s, it was gently guided to the platform and kept there for 10 s. After the trial, each mouse was placed in a holding cage under a warming lamp for 25 s until the start of the next trial. For the probe trials, the mice were allowed to swim freely in the pool for 60 s with platform removal. The escape latency, the time spent in the opposite quadrant to the platform zone, and the frequency in the platform zone were measured.

### 4.6. Passive Avoidance Test

The experimental procedure involved the examination of memory acquisition. An increase in training latency and decline in retention latency signified impaired memory in the task [74,75]. The passive avoidance test was carried out as one-trail step-through using the apparatus that consisted of a two-compartment acrylic box with a light compartment and a dark one equipped with an electric grid floor (Ugo Basile SRL, Varese, Italy). At the beginning of the training session, mice were placed in the light compartment, a condition that makes the animals uncomfortable. After 60 s the door between the two compartments was opened and the entrance of the animals to the dark compartment was punished by an electric foot shock (0.2 mA for 2 s). The latency time for entering the dark compartment was recorded during both the training day and the trail day, which was provided 24 h after the training trial, without any electric shock. On the test day, mice were placed in the light compartment and after 60 s the door was opened for 6 minutes, the latency time for entering in the compartment of preference was recorded. 

### 4.7. Tissue Preparation for Immunohistochemistry and Neurochemical Analysis

Ten and 20 days after Aβ_1–42_O injection, mice were deeply anesthetized and sacrificed by cervical dislocation. The brains were removed and the left hemisphere of each animal was immersed in a 4% fixative solution of paraformaldehyde (Santa Cruz Biotechnology, Dallas, TX, USA) for 48 h. Fixed brains were sliced on a vibratome (Leika Microsystems, Milan, Italy) at 40 μm thickness. 

Right hemispheres were rapidly removed, and the hippocampi were dissected in an ice-cold plastic dish and homogenized as previously described [6]. Proteins in the nuclear fraction were extracted using a Nuclear Extract kit (Actif Motif, Carlsbad, CA, USA). Cytoplasmic and nuclear protein concentrations were determined by the Bradford assay [76].

### 4.8. H&E Staining

The H&E staining was performed as described by Fischer et al. [77]. Briefly, selected sections were mounted on slides and dried before dipping them in 100%, 95% and 70% ethanol (Sigma-Aldrich, Saint Louis, MO, USA). Slices were washed and stained with hematoxylin for 8 min and then incubated for 10 min in tap water to promote the change to violet coloration. Subsequently, slices were washed in distilled water and then dipped 10 times in 80% ethanol before being immersed in 25% eosin solution (in ethanol 80%) for 1 min. Finally, slices were dehydrated in 95% and 100% ethanol solutions for 5 min before being fixed in xylen. Image analysis was performed by a blinded investigator, using an AxioImager M1 microscope (Carl Zeiss, Oberkochen, Germany) and a computerized image analysis system (AxioCam MRc5, Carl Zeiss) equipped with dedicated software (AxioVision Rel 4.8, Carl Zeiss). After defining the boundary of the hippocampus at low magnification (2.5× objective), H&E staining was evaluated by densitometry of five different sections for each sample analyzed at a higher magnification (20× or 40× objective). Quantification and morphological analysis were performed with the ImageJ software. 

### 4.9. Determination of Caspases Activation

Caspase-3 and -9 enzyme activities were determined using a protocol adapted by Movsesyan et al. [78]. Briefly, samples were incubated with the assay buffer (20% sucrose; 50 mmol/L Hepes, pH 7.4; 2 mmol/L EDTA; 0.2% CHAPS; and 10 mmol/L dithiothreitol) and a 50 mmol/L concentration of chromogenic pNA specific substrates (Z-Asp-Glu-Val-Asp-pNA for caspase-3 and Ac–Leu–Glu–His–Asp–pNA for caspase-9; Alexis Biochemicals, San Diego, CA, USA). In a final volume of 100 μL (containing 60 μg of proteins), each test sample was incubated for 3 h at 37 °C. The amount of chromogenic pNA released was measured with a microplate reader (GENios, TECAN^®^, Mannedorf, Switzerland) at 405 nm. 

### 4.10. Determination of Redox Status

The redox status was measured as described previously, by the detection of ROS formation [24]. ROS were measured using the fluorescent dye 2′,7′-dichlorodihydrofluorescein diacetate (DCFH-DA). In the presence of ROS, nonfluorescent DCFH-DA converts to fluorescent 2′,7′-dichlorofluorescein (DCF), which is read using a microplate reader (GENios, TECAN^®^). The fluorescence emission intensity of DCF (535 nm) was detected in response to 485 nm excitation. Background fluorescence was corrected by the inclusion of parallel blanks. 

### 4.11. Determination of GSH Content

GSH content was measured as described previously [13]. Briefly, samples were first deproteinized with 4% of sulfosalicylic acid, centrifuged to remove the precipitated protein, and then assayed for glutathione with 5-5′-dithiobis (2-nitrobenzoic acid) (4 mg/mL in phosphate buffer, 0.1 M, pH 7.4). The yellow color that developed was read immediately at 412 nm (GENios, TECAN^®^). Results were calculated using a standard calibration curve. 

### 4.12. Determination of Nrf2 Activation

The Nrf2 activation was assessed as described previously by Morroni et al. [29] using an ELISA kit (TransAM^®^ Kit, Active Motif) according to manufacturer’s instructions. The assay consisted of an immobilized oligonucleotide containing the ARE consensus-binding site (5′-GTCACAGTGACTCAGCAGAATCTG-3′) that was used to measure the binding activity of Nrf2 to the DNA. Nrf2 from the nuclear extract samples was allowed to bind to the ARE on 96-well plates. A primary antibody against Nrf2 was then used to detect bound Nrf2. After the incubation with a secondary antibody conjugated to HRP, the plate readout was 450 nm with a reference length at 655 nm (GENios, TECAN^®^). Results were normalized to the protein concentration in each sample. 

### 4.13. Western Blotting

Samples (30 μg proteins) were separated on 12% SDS polyacrylamide gels (Invitrogen, Carlsbad, CA, USA) and electroblotted onto 0.2 μm nitrocellulose membranes. Membranes were incubated overnight at 4 °C with primary antibody recognizing Phospho-GSK3β (Ser21/9) or Phospho-p44/42 MAPK (ERK1/2) (Thr202/Tyr204) (1:1000; Cell Signaling Technology Inc., Danvers, MA, USA). Membranes were then incubated with a horseradish peroxidase linked anti-rabbit secondary antibody (1:2000; GE Healthcare, Piscataway, NJ, USA). Immunoreactive bands were visualized by enhanced chemiluminescence (ECL; Pierce, Rockford, IL, USA). The same membranes were stripped and reprobed with total GSK3β or total p44/42 MAPK (1:1000; Cell Signaling Technology Inc.), respectively. Finally, to obtain a further loading control, membranes were stripped and reprobed with a monoclonal primary antibody recognizing β-actin (1:1000; Sigma-Aldrich) and then with a horseradish peroxidase linked anti-mouse secondary antibody (1:2000; GE Healthcare). Data were analyzed by densitometry, using Quantity One software (Bio-Rad, Hercules, CA, USA).

### 4.14. GFAP and Iba1 Staining

After deparaffinization, slices were washed in PBS and then incubated in tris-buffered saline (TBS)-A (0.1% Triton X-100 solution) and then TBS-B (TBS-A 2% BSA) to minimize non-specific absorption. Sections were then incubated overnight at 4 °C with a mouse anti-GFAP primary antibody (1:300; Cell Signaling Technology Inc.) and a rabbit anti-Iba1 primary antibody (1:300; Wako Pure Chemical Industries, Osaka, Japan) in TBS-B with 3% Normal Goat Serum (NGS, Wako Pure Chemical Industries). Twenty-four hours later, slices were washed with TBS-A and TBS-B before the incubation with secondary anti-rabbit antibody (1:200; Alexa Fluor^®^ 555, Life Technologies) and secondary anti-mouse antibody (1:200; Fluorescein, Life Technologies) in TBS-B with 3% NGS. The binding specificity was verified by the incubation of some sections with only primary or secondary antibodies, and no positive staining was found, indicating that the immunoreactions were positive in all experiments carried out. A blinded image analysis was performed using an AxioImager M1 microscope (Carl Zeiss) and a computerized image analysis system (AxioCam MRc5, Carl Zeiss) equipped with dedicated software (AxioVision Rel 4.8, Carl Zeiss). After defining the boundary of the hippocampus at low magnification (2.5× objective), GFAP and Iba1 staining were evaluated by densitometry of five different sections for each sample analyzed at a higher magnification (20× or 40× objective). Quantification and morphological analysis were performed with the ImageJ software. 

### 4.15. RNA Extraction

Total RNA from the hippocampus was isolated using Pure link RNA mini kit (Thermo Fisher Scientific, Life Technologies, Carlsbald, CA, USA), according to the manufacturer’s instructions. Briefly, hippocampal samples were homogenized in Lysis buffer with 1% β-mercaptoethanol by a homogenizer SHM1 (Stuart, Bibby Scientific LTD, Staffordshire, UK) on ice. Homogenized samples were added to an equal volume of 70% ethanol and mixed. The solution was passed through a filter cartridge, containing a clear silica-based membrane to which the RNA binds, and washed with Wash Buffer I and Wash Buffer II. RNA was finally eluted with RNase-free water and stored at −20 °C.

#### RNA Reverse Transcription and Real Time PCR

Before reverse transcription, RNA was quantified using Nanoquant plate (TECAN). For each sample, 200 ng of total RNA were reverse transcribed using the High Capacity cDNA Reverse Transcription kit (Life Technologies), according to the manufacturer’s recommendations. Briefly, 10 µL of the sample were added to 10 µL master mix with RNase inhibitors and subjected to the appropriate thermocycling conditions. Finally, relative quantification by Taqman gene expression assay (Life Technologies) was performed by real time PCR (BIORAD CFX Connect) for the following gene: NOS2 (Mm00440502_m1) and as well as rn18S (Mm03928990_g1) and ACTB (Mm00607939_s1), as endogenous controls, using Universal Master Mix (Life Technologies). Each measurement was performed in triplicate and data were analyzed through the 2^−ΔΔCt^ method [79]. Sham mice were considered the calibrator of the experiment. 

### 4.16. Statistical Analysis

Data were analyzed with the PRISM 5 software (GraphPad Software, La Jolla, CA, USA) and expressed as a fold increase ± SEM of each group compared to the Sham/VH group. The difference between groups was analyzed using one-way ANOVA with a Bonferroni post hoc test. A difference was considered statistically significant when a *p* value was less than 0.05.

## Abbreviations

6-MSITC	6-(Methylsulfinyl)hexyl isothiocyanate
Aβ_1-42_O	β-Amyloid oligomers
AP-1	Activator protein-1
AD	Alzheimer’s disease
ARE	Antioxidant responsive element
JNK	c-Jun N-terminal kinases
COX-2	Cyclooxygenase-2
CNS	Central Nervous System
DCF	2′7′-dichlorofluorescein
DCFH-DA	2′7′-dichlorodihydrofluorescein diacetate
DNA-PK	DNA-Dependent Protein Kinase
ERK12	Extracellular signal-regulated protein kinases 1 and 2
GFAP	Glial Fibrillary Acidic Protein
GSH	Glutathione
GSK3	Glycogen synthase kinase 3
H&E	Hematoxylin-Eosin
Iba1	Ionized calcium-binding adaptor molecule 1
i.c.v.	Intracerebroventricular
iNOS	Inducible Nitric Oxide Synthase
i.p.	Intraperitoneal
ITCs	Isothiocyanates
Keap1	Kelch-like ECH-associated protein 1
MAPK	Mitogen-activated protein kinase
Morris Water Maze	MWM
NGS	Normal Goat Serum
NO	Nitric Oxide
Nrf2	Nuclear factor E2-related factor 2
PARP	Poly ADP Ribose Polymerase
PBS	Phosphate Buffer Saline
pNA	p-nitroaniline
ROS	Reactive Oxygen Species
TBS	Tris-buffered saline
VH	Vehicle
